# Spatial-frequency dependent binocular imbalance in amblyopia

**DOI:** 10.1038/srep17181

**Published:** 2015-11-25

**Authors:** MiYoung Kwon, Emily Wiecek, Steven C. Dakin, Peter J. Bex

**Affiliations:** 1 Department of Ophthalmology, School of Medicine, University of Alabama at Birmingham, Birmingham, AL; 2 Department of Psychology, Northeastern University, Boston, MA; 3 New England College of Optometry, Boston, MA; 4 UCL Institute of Ophthalmology, University College London, UK; 5 School of Optometry and Vision Science, University of Auckland, New Zealand.

## Abstract

While amblyopia involves both binocular imbalance and deficits in processing high spatial frequency information, little is known about the spatial-frequency dependence of binocular imbalance. Here we examined binocular imbalance as a function of spatial frequency in amblyopia using a novel computer-based method. Binocular imbalance at four spatial frequencies was measured with a novel dichoptic letter chart in individuals with amblyopia, or normal vision. Our dichoptic letter chart was composed of band-pass filtered letters arranged in a layout similar to the ETDRS acuity chart. A different chart was presented to each eye of the observer via stereo-shutter glasses. The relative contrast of the corresponding letter in each eye was adjusted by a computer staircase to determine a binocular *Balance Point* at which the observer reports the letter presented to either eye with equal probability. Amblyopes showed pronounced binocular imbalance across all spatial frequencies, with greater imbalance at high compared to low spatial frequencies (an average increase of 19%, *p* < 0.01). Good test-retest reliability of the method was demonstrated by the Bland-Altman plot. Our findings suggest that spatial-frequency dependent binocular imbalance may be useful for diagnosing amblyopia and as an outcome measure for recovery of binocular vision following therapy.

Binocular vision, in which the two eyes work together, supports not only depth perception^1^, but also a variety of complex visual tasks such as reading, object recognition, and visual-motor coordination^2^,^3^,^4^. When normal binocular vision is disturbed by ocular misalignment (strabismus), unequal refractive errors (anisometropia), or any other condition producing unequal binocular input (e.g. cataract), the visual system is at risk of developing amblyopia. Amblyopia is an optically uncorrectable loss of vision, usually in one eye, without any known pathology^5^. It is the most common cause of monocular visual loss in children, affecting approximately 3–5% of the population^6^ and is associated with a range of deficits in monocular and binocular visual function^7^,^8^,^9^,^10^,^11^.

Accumulating evidence indicates that abnormal binocular interaction plays a key role in the etiology of amblyopia^12^,^13^. Amblyopic binocular vision is characterized by a weakening of the excitatory connections supporting binocular summation and stereopsis^14^,^15^,^16^, and a strengthening of the inhibitory connections supporting interocular suppression^17^. Stronger suppression is associated with a more severe amblyopic deficit^11^,^12^,^18^,^19^,^20^,^21^,^22^,^23^. Importantly, when suppression is alleviated by equating the effective contrast of the two eyes (i.e., binocularly balanced stimuli), some amblyopes are able to achieve binocular fusion, possibly via a strengthening of the excitatory binocular connections supporting binocular summation^11^,^12^,^19^,^21^,^24^,^25^,^26^,^27^. Recent treatment regimens designed to reduce suppression by promoting an exposure to binocularly balanced stimuli have been shown to improve visual acuity and stereoacuity^28^,^29^,^30^,^31^. Collectively, these observations suggest that addressing the imbalance in monocular signals may restore normal binocularity in amblyopia. Consequently, assessment of abnormal binocular interaction will likely assume an increasingly important role in both the detection and treatment of amblyopia.

A considerable literature indicates that core amblyopic deficits such as contrast sensitivity loss and spatial distortion exhibit spatial-frequency dependency. For example, contrast sensitivity loss in amblyopia is more pronounced at mid-to-high spatial frequencies (SFs), with deficits at low SFs being less common^7^,^14^,^32^,^33^,^34^,^35^,^36^, although some amblyopes show deficits in all spatial frequencies^7^. Similarly, perceptual distortion is more severe at higher SFs, with low SFs being perceived veridically^37^,^38^. On the other hand, Hess and Bradley^39^ reported that amblyopia does not influence supra-threshold apparent contrast at any SF, suggesting that gain control mechanisms compensate for deficits at detection threshold^40^. This distinction between threshold and supra-threshold contrast perception in amblyopia raises fundamental questions concerning the SF dependence of binocular interactions. A study completed by Ding *et al.*^23^ indeed indicated a potential dependence of binocular interactions on SF. Taken together, these studies suggest that abnormal binocular interaction may be differentially affected by different SFs. However, no clinical assessment is currently available for quantifying such SF-dependent binocular imbalance.

The primary goal of the current study is to assess binocular imbalance as a function of spatial frequency in amblyopia using a novel computer-based method. To assess the effect of SF on binocular imbalance, we developed a novel dichoptic letter chart that is illustrated in the Method section. To ensure familiarity with the stimuli, we used SF band-pass filtered Sloan letters that were arranged in a layout similar to the ETDRS acuity chart (4 rows of decreasing letter size by 5 columns of varying letter contrast) on a gray background. A different letter chart is presented to each eye of an observer via stereo-shutter glasses. At each position, the identity and interocular contrast-ratio of the letter on each chart differs while the spatial-frequency content of the letter remains the same. Participants were instructed to read aloud the chart in top-to-bottom and left-to-right order. The relative contrast of the letter in each eye is adjusted across several subsequent charts to determine *the balance* point (BP) between the two eyes: the interocular contrast-ratio required to report the letter in each eye with equal probability. Therefore, a BP of 0.5 indicates that the input signals from the two eyes are equated whereas a value that is significantly different from 0.5 suggests binocular imbalance.

In this study, we use the term interocular suppression interchangeably with binocular imbalance or abnormal binocular interaction to refer to a functional imbalance between the two eyes in a broader sense. This comprehensive approach was taken based on the following reasons: 1) previous studies have demonstrated that not only inhibition of the amblyopic eye from the fellow eye, but also attenuation of input signals in the amblyopic eye contributed to the deficit in amblyopia^19^,^23^. Thus, it is important not to restrict our definition of amblyopic deficit only to inhibitory suppression; 2) in order to measure overall binocular imbalance, we took advantage of the known sensory phenomenon of binocular rivalry by equating the input contrast signals from the two eyes. Binocular rivalry occurs when the two eyes view markedly different stimuli: the observer perceives only one stimulus at a time, and perception alternates between the two stimuli at irregular intervals^41^,^42^,^43^. Previous research has shown that under conditions of binocular rivalry, normally-sighted observers perceive the image presented to the dominant eye (as determined by a hole-in-card test) sooner and for longer than images presented to the non-dominant eye^44^,^45^,^46^. Furthermore, for visually-impaired observers, dominance shifts towards the less impaired eye when contrast sensitivity is reduced by cataract^47^. The important and relevant proposition of binocular rivalry is that increasing stimulus strength (e.g., luminance contrast) for one eye increases the perceptual dominance (i.e., the total proportion of the binocular rivalry viewing time that a stimulus is dominant) of that eye’s stimulus^43^. In other words, our present method measures the balance point where the two eyes’ stimulus strength reaches equilibrium so that the proportion of the viewing time for the two eyes is approximately the same, i.e., 0.5. While the exact neuronal mechanism of binocular rivalry or interocular suppression remains unclear, it has been suggested that the underlying mechanisms of binocular fusion, stereopsis and rivalry are closely related to each other or at least partly based on a common mechanism^48^,^49^,^50^,^51^,^52^. Therefore, our present method aims to provide a quantitative measure of the balance point between the two eyes in amblyopia with a repeatable test that is simple to administer in the clinic.

## Results

### Binocular imbalance at high SFs in amblyopia

**Figure 1:**
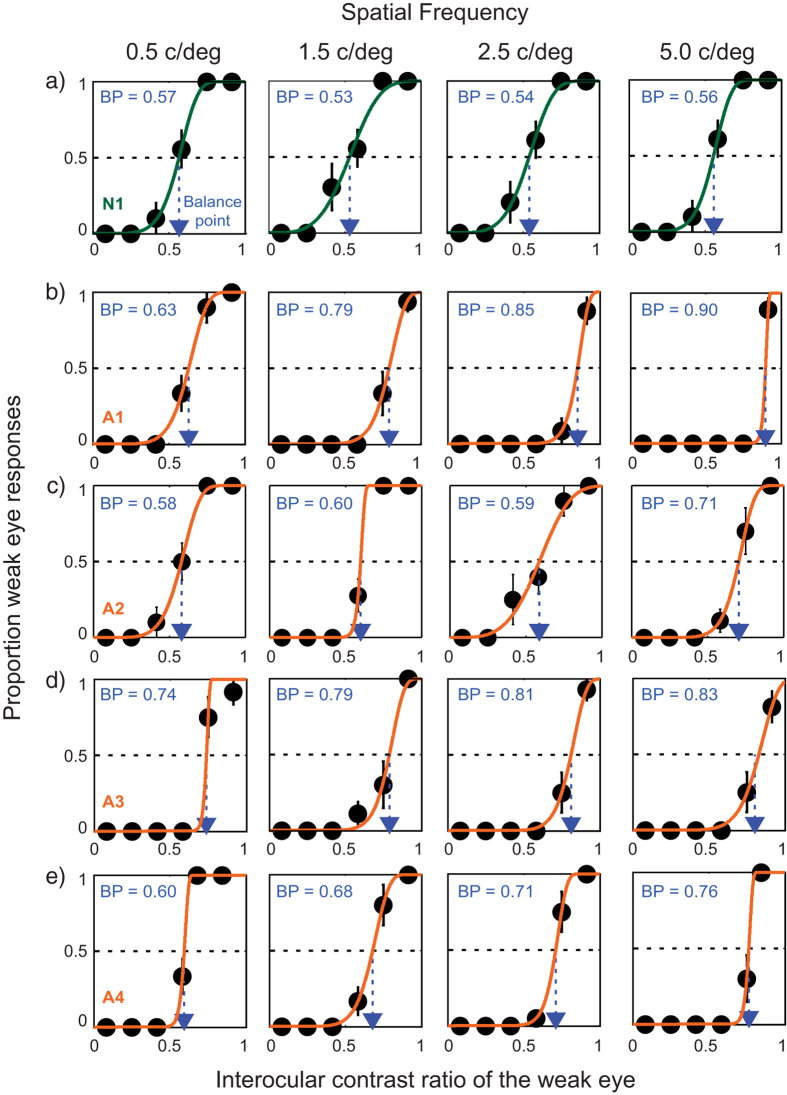
Examples of participants’ data. Each panel shows the proportion of trials when the observer reported the identity versus interocular contrast ratio for a representative subject from each group. Each column represents the data for each spatial frequency (0.5, 1.5, 2.5, and 5 c/deg). The BP of the weak eye is also shown in the caption for each panel. The data (black circles) were fitted with a Weibull function to estimate the BP of the weak eye. The solid lines are the best fits of the model. The dotted arrow lines (blue) indicate estimated BPs. **(a)** An individual with normal vision (N1); **(b-e)** Individuals with anisometropic amblyopia (A1–A4).

Figure 1 plots the proportion of trials on which the observer reported the letter presented to their weaker eye against interocular contrast ratio. Each row shows data from an individual participant: top row for a participant with normal vision (N1) and the bottom three rows for four representative participants with amblyopia (A1, A2, A3, A4). Each column indicates the data for each spatial frequency (0.5, 1.5, 2.5 and 5 c/deg). The balance point (BP) was estimated by fitting the data with a Weibull function and finding the contrast ratio corresponding to a 0.5 proportion of weak eye responses. Overall, the model fits were satisfactory with *r*^*2*^ values of 0.86 to 0.99 (mean 0.99 ± 0.02), indicating that about 99% of variance is accounted for by the Weibull model.

The BPs of participants with amblyopia increased with increasing spatial frequency (SF), which manifest as a steadily increasing rightward shift of the psychometric function at higher SFs. For example, the BP of the weak eye (A1 in Fig 1b) increased from 0.63 at 0.5 c/deg. to 0.90 at 5 c/deg. These data indicate that for a low SF, 63% contrast is required for the weak eye to balance 37% contrast in the strong eye while for a high SF 90% of contrast is needed for the weak eye to balance 10% contrast in the strong eye. This substantially higher BP was observed in all participants with amblyopia suggesting that input from the weak eye is attenuated or suppressed by the strong eye, even under conditions of suprathreshold perception. This suppression is more pronounced at higher SFs, even though all stimuli were viewed foveally. The BPs of normally sighted observers were close to a value of 0.5, indicating that the input signals from the two eyes are treated approximately equally and in a manner that is largely independent of SF.

Note that this difference in BP between SFs is a result of a binocular interaction and cannot be being driven by amblyopic observers being simply unable to resolve high SF stimuli. We know this because the psychometric function derived from amblyopic observers presented in Fig. 1 reach close to 1.0 on the ordinate, i.e. provided the interocular contrast ratio of the weaker eye is high enough, all observers correctly report close to 100% of the stimuli presented to the weaker eye.

**Figure 2:**
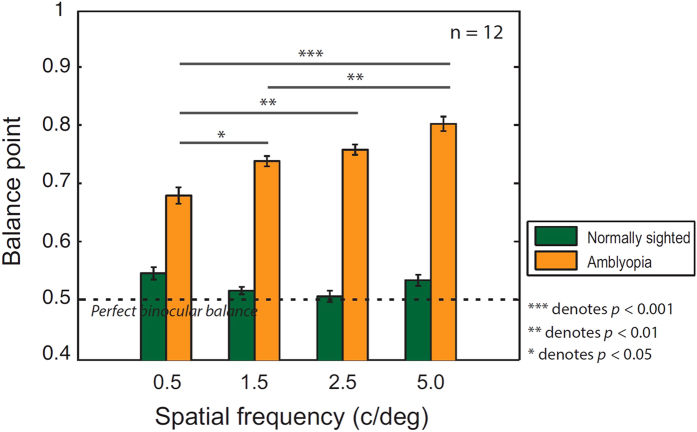
Mean balance points for normally-sighted and amblyopic observers at four spatial frequencies. The mean BPs as a function of SF were plotted for both amblyopic (orange bars; *n* = 12) and normally-sighted control (green bars; *n* = 12) groups. The dotted line indicates a value of 0.5 where the input signals from the two eyes are treated equally (i.e. perfect binocular balance). Error bars represent  ±1 Standard Errors of the Mean (SEM).

Figure 2 shows the mean BPs as a function of SF for the two participant groups: amblyopic (orange bars) and control (green bars). The dotted line indicates a proportion of weak-eye responses equal to 0.5, indicating balanced contrast perception between the two eyes. Consistent with individual data (Fig. 1), we observed that across all the SFs, the BPs for the amblyopic group (0.75 ± 0.13) were considerably higher than for the normal control group (0.53 ± 0.05). It is worth noting that the average BP of the normal control group was slightly greater than a value of 0.5, indicating moderate eye dominance for the dominant eye in normally-sighted individuals.

A two-way repeated measures ANOVA showed a significant main effect of subject group (*F*_(1, 88)_ = 122.28, *p* < 0.001) on BP. We also found that the BP of the amblyopia group significantly differed across different SFs (*F*_(3, 33)_ = 14.11, *p* < 0.001) while that of the normal control group remained constant across different SFs (*F*_(3,33)_ = 2.55, *p* = 0.073). Tukey’s HSD pairwise comparison test further revealed that the BP of each SF was significantly different from each other except for two pairs (1.5 SF vs. 2.5 SF; 2.5 SF vs. 5 SF) in the amblyopia group. Importantly, the BP of the SF of 0.5 c/deg was significantly lower than that of 1.5, 2.5 or 5 c/deg (all *p* < 0.05) whereas the BP of the SF of 5 c/deg was significantly higher than that of 0.5 c/deg (*p* < 0.001) or 1.5 c/deg (*p* = 0.011), suggesting that the BP increases with SF in amblyopic observers.

**Figure 3:**
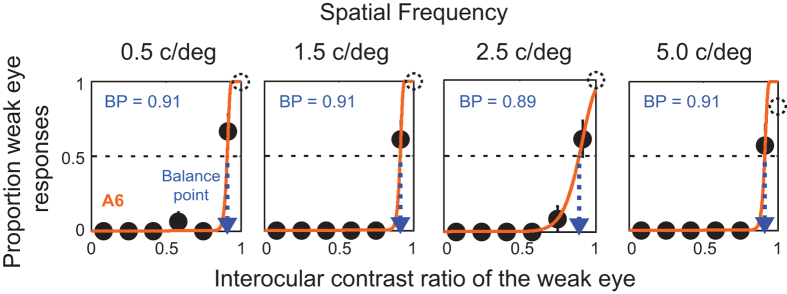
An amblyopic observer whose binocular imbalance was not dependent on spatial frequency. Each panel contains the proportion of weak eye’s letter identity responses versus interocular contrast ratio for a subject (A6) whose binocular imbalance was not affected by spatial frequency. The BPs of this subject were consistently high (~0.9) across all four spatial frequencies. The data (solid black circles) were fitted with a Weibull function to estimate the binocular BP. The solid lines are the best fits of the model. The dotted arrow lines (blue) indicate estimated BPs. The black open circles are correct identification of letters presented to the weaker eye with an interocular contrast ratio of 1.0 (i.e. no signal to the strong eye).

It is noteworthy that while the vast majority of amblyopic observers showed spatial-frequency dependent binocular imbalance, there were a couple of amblyopic observers (2 out of 12: A5 and A6) who showed no dependency of binocular imbalance on spatial frequency (Fig. 3). As shown in Fig. 3, the BPs of this observer remained consistently high (~0.90) across all spatial frequencies. These observed individual differences further highlight the importance of assessing spatial-frequency dependent binocular imbalance on an individual basis. We note that these observers achieved near 100% accuracy for the letters (shown by the black open circles) presented to the weaker eye when its interocular contrast ratio was 1.0 (i.e. no signal to the strong eye). Thus it is not likely that a SF-broadband acuity-deficit contributed to the results from these participants. These participants’ interocular acuity difference (0.4 logMAR for A5; 0.1 logMAR for A6) and steroacuity (400″ for A5; 900″ for A6) did not systemically differ from the rest of the participants. It might be simply because the depth of amblyopia was greater in these two subjects compared to other amblyopes. While it still remained unclear what factors play a major role in our results from these participants, our finding might relate to the fact that there are two classes of amblyopes: one class has contrast sensitivity deficits in only high and intermediate spatial frequency; the other has deficits in all spatial frequencies including low spatial frequencies^7^.

### Test-retest reliability of the binocular imbalance assessment method

To examine whether our test provides a stable estimate of binocular imbalance, a subset of participants performed the test twice (16 out of 24 subjects participated this test: 6 amblyopes and 10 normally-sighted individuals). Test-retest reliability was evaluated using i) Pearson’s Product Moment Correlation coefficient (*r*) and ii) Bland & Altman difference plot^53^ in which difference values between the two measurements (i.e., 1^st^ test − 2^nd^ test) are plotted as a function of mean values of the two (i.e., (1^st^ test + 2^nd^ test)/2) for each subject. A value of zero on the y-axis in a Bland & Altman plot indicates no change between two tests while larger deviation from the value of zero means larger variability between tests. The 95% limits of agreement show the range within which a difference value between tests is likely to fall for most participants (95%).

**Figure 4:**
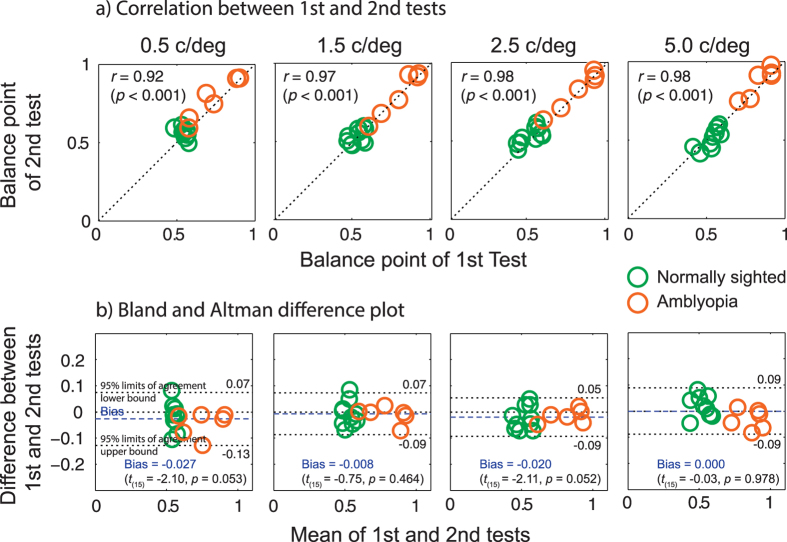
Test-retest reliability. **(a)** Correlation between 1^st^ and 2^nd^ tests. The dotted lines indicate the line of equality (1^st^ test = 2^nd^ test). Each circle indicates a data point from one subject, orange for amblyopic observers, green for normally-sighted controls; **(b)** Difference in BP between 1^st^ test and 2^nd^ test as a function of mean value of the two tests. Each circle indicates a data point from each subject, orange for amblyopes, green for controls. The horizontal blue dotted lines represent a bias of the test, i.e., the mean difference value across participants. The horizontal black dotted lines represent 95% limits of agreement.

Figure 4a shows a plot of the BP values of the second test against those of the first. The dotted lines indicate the line of equality, where the BP value of first test is the same as that of second test across participants. There was excellent agreement between the two measurements indicated by Pearson’s correlation coefficients ranging from *r* = 0.92 (*p* < 0.001) to *r* = 0.98 (*p* < 0.001). Figure 4b shows that there was no significant bias indicated by the Bland and Altman difference plot. In other words, the observed mean difference value of −0.027 for the SF of 0.5 c/deg represented as the horizontal blue solid line in Fig. 4b was not statistically different from the value of zero and this pattern of results was consistent for each of the SF conditions (*all p* > 0.05). The test-retest variability was estimated as 95% confidence interval limits of agreement (mean difference between measures ± 1.96 SD). The mean difference between 1^st^ and 2^nd^ tests for the SF of 0.5 c/deg was −0.027 (95% CI, −0.13 to 0.07); for 1.5 c/deg it was −0.008 (95% CI, −0.09 to 0.07); for 2.5 c/deg it was −0.020 (95% CI, −0.09 to 0.05); and for 5 c/deg it was −0.001 (95% CI, −0.09 to 0.09). Thus the BP differences between the two measures all fell within less than a mean difference of 0.09 (<~9%), suggesting reasonably good test-retest reliability.

## Discussion

A number of studies have shown that core amblyopic deficits such as contrast sensitivity loss and spatial distortion are more marked at high SFs than at low SFs^32^,^33^,^34^,^35^,^36^, suggesting a significant role of SF in amblyopic vision. However, little is known about whether binocular imbalance is modulated by the SF component of stimuli (e.g., the scale of an object). Recent evidence demonstrates that suppression is a key factor in the etiology of amblyopia^12^,^13^, as well as a potential target for novel therapeutic interventions. Adequate assessment of binocular imbalance and its dependence on SF may provide an additional, sensitive diagnostic tool for amblyopic vision that may help in the design of more effective treatment regimens. Indeed, recent studies have shown the clinical value of the quantitative assessment of binocular imbalance in managing individual patients’ treatment outcomes and prognosis^20^,^22^,^28^. Unfortunately, currently available clinical tests such as the Worth 4 dot, the Bagolini striated lenses and OXO tests provide only a binary classification of suppression versus no suppression. None of these outcomes provide a quantitative estimate of binocular imbalance or its remediation during treatment. Moreover, they do not offer any detailed information with respect to SF. The current study proposes a quantitative and clinically viable method of assessing binocular imbalance and its SF-dependence in amblyopia. We developed a novel dichoptic letter chart that estimates the contrast balance between the two eyes. The *Balance Point* was defined as the interocular contrast ratio at which the percepts of the two eyes reach equilibrium. The BP was estimated over multiple spatial scales using a sequence of letter charts via an adaptive procedure. The observer’s task is simply to read the letters on the chart out loud. Our novel method was designed to efficiently assess the magnitude of binocular imbalance on a wide range of spatial scales ranging from fine (e.g., 5 c/deg) to coarse scales (e.g., 0.5 c/deg), while maintaining an interface that is familiar to patients and easy to use for clinicians.

With this method, we found evidence that binocular imbalance in the great majority of amblyopic observers (10 out of 12) was strongly dependent on SF: binocular imbalance was stronger at higher SFs than lower SFs (an average increase of 19%). This pattern of results is in a good agreement with the findings of Ding *et al.*’s study^23^ showing that when the SF of a grating increased from 0.68 to 2.72 c/deg, the contrast in the strong eye had to be reduced more at higher SFs in order to achieve binocularly balanced vision.

In addition, we also observed that for a given SF, the amblyopic eye requires a higher contrast to balance the fellow eye (75% for the amblyopic eye vs. 53% for the non-dominant eye of normally-sighted individuals, on average across all SFs). Our results are in good agreement with the findings of recent binocular interaction studies showing abnormal binocular interaction in amblyopia. Despite the differences in the methodology employed to assess the interocular imbalance in amblyopic vision^11^,^12^,^18^,^19^,^20^,^21^,^22^,^23^, these studies consistently showed that the contrast signal in the weak eye of amblyopic observers was considerably and consistently attenuated relative to that in the strong eye. For example, Huang *et al.*^11^ found that the effective contrast ratios of the weak eye ranged from 0.11 to 0.28, indicating that only 11% to 28% of contrast is required for the strong eye to match 100% contrast in the weak eye. Similarly, Kwon *et al.*^18^ showed that amblyopes exhibited significantly reduced effective contrast (~20%) of the weak eye, compared to normally sighted controls.

What might be the underlying mechanism for spatial-frequency dependent binocular imbalance?

Is it simply due to an acuity-deficit in the amblyopic eye (i.e., difficulty recognizing smaller letters)? We do not think this is the case. As demonstrated in Fig. 1 and 3, we found that all amblyopic observers correctly reported the letter identity of the stimuli presented to the weak eye (close to 100% accuracy) when the interocular contrast ratio of the weak eye was high enough (especially when no signal was given to the strong eye). These results indicate that pronounced binocular imbalance for high spatial-frequency stimuli is likely to reflect active suppression of the monocular response to the weak eye rather than any monocular acuity deficit per se.

A large body of work suggests that amblyopia is a cortical phenomenon^54^,^55^, likely originating in primary visual cortex^56^,^57^,^58^. Most neurons in visual cortex receive binocular input and these neurons are most sensitive to stimuli of a specific SF and orientation falling within their receptive field^59^. The cortical arrangement of SF preference is a matter of ongoing debate^60^,^61^,^62^,^63^,^64^ but there is evidence that the high and low SFs are mapped onto different areas of ocular dominance columns, with low SF selective neurons tending to lie in the center of columns whereas high SF selective neurons being found at the boundaries between left and right eye ocular dominance columns^65^,^66^. When animals are deprived of binocular vision during development, they exhibit abnormally narrow ocular-dominance stripes for the deprived eye and abnormally wide stripes for the fellow eye^67^,^68^,^69^. Barrett *et al.*^37^, conjectured that this reorganization of the ocular-dominance map in amblyopia should selectively affect vision of higher SFs because the expansion of the neural territory dominated by one eye at the expense of the other eye occurs first at the boundary between ocular-dominance columns, which happens to be the location of the high-frequency component of the SF map. This hypothesis is corroborated by neurophysiological evidence showing that binocular interactions in humans with strabismic amblyopia are SF dependent^70^. Consistent with this hypothesis, Barrett *et al.*^37^ found that spatial distortion becomes more pronounced at higher SFs in amblyopia while errors in perception were less obvious with stimuli at low SFs (≤1.25 c/deg).

If this were the case, we should expect to see a similar SF dependent loss measured by the contrast sensitivity function (CSF). Consistent with this prediction, a large number of studies have reported that contrast sensitivity loss in amblyopia is spatial-frequency dependent. The CSFs of amblyopic vision show moderate to severe deficits which tend to be more marked at mid and high SFs^7^,^32^,^33^,^34^,^35^,^36^ with less pronounced deficits at low SFs. Levi, Harwerth and Smith^14^ reported observers with abnormal binocular vision have binocular interactions that are narrowly tuned to the SF and orientation of the stimulus. For example, a suprathreshold masking grating presented to one eye elevated the contrast threshold for gratings presented to the fellow eye, within a narrow range of SFs (about 1 octave width at half height) and orientation, centered about the SF and orientation of the mask.

While contrast sensitivity loss in mid and high SFs appears to be prevailing in amblyopes, Hess and Howell^7^ demonstrated that some amblyopes exhibit deficits in all spatial frequencies including low SFs, suggestion two different classes of amblyopes. Interestingly, we also observed that two out of twelve amblyopes show consistently high binocular imbalance across all SFs. This might be simply due to the depth of amblyopia being greater in these two amblyopes than the others. Alternately, our findings might have reflected the existence of these two classes of amblyopes, further stressing the importance of assessing spatial-frequency dependent binocular imbalance in amblyopia. However, the exact underlying mechanism still remains to be addressed for the future study.

Our data are consistent with the foregoing studies showing the presence of spatial frequency-dependent contrast sensitivity deficits in amblyopia and extend our understanding to the perception of binocular contrast at supra-threshold levels (whether it involves presentation of the same or different stimuli to each eye). We show that binocular imbalance is greater at high SFs than at low SFs for a majority of amblyopes, suggesting a complex binocular interaction in spatial vision. New and emerging binocular therapies for amblyopia are directly addressing binocular imbalance^28^,^29^,^30^,^31^, but currently lack an efficient and quantitative assessment of binocular imbalance. We speculate that the quantification of binocular imbalance may provide a valuable clinical outcome measure of visual impairment in amblyopia that may be important for diagnosis and prognosis. Particularly, the assessment of SF-dependent binocular imbalance may assist clinicians or vision rehabilitation specialists in choosing optimal treatment strategies and outcome measures suited for individual patients, thereby improving the management of amblyopic vision.

## Methods

### Participants

A total of 24 participants took part in this study: 12 control individuals with normal or corrected-to-normal vision (mean 27.80 ± 7.89 years; 6 males; mean binocular visual acuity −0.13 ± 0.09 logMAR) and 12 individuals with amblyopia (mean 24.75 ± 7.85 years; 3 males). The amblyopia group included 1 strabismic amblyopes, 1 mixed (anisometropic and strabismic) amblyope, and 10 anisometropic amblyopes. Amblyopia was defined as worse than or equal to 0.00 logMAR best-corrected visual acuity in amblyopic eye with poorer than or equal to 400 arcsec stereoacuity and the presence of suppression (a Worth 4 dot test). Anisometropia was defined as >0.50 diopter (D) of spherical equivalent and/or >1.50 D difference between the eyes in astigmatism. Normal vision was defined as better than or equal to 0.00 logMAR best-corrected visual acuity in each eye with normal stereoacuity and without known visual disorder. Participants with any known cognitive or neurological impairments were excluded from the study.

The experimental protocols and procedure were approved by the institutional review board of Boston Children’s Hospital or the institutional review board of University of Alabama at Birmingham. The protocols and procedure complied with the Declaration of Helsinki, and written informed consent was obtained from all participants prior to the experiment. The mean age, best corrected visual acuity (ETDRS charts), refractive error, angular eye deviation (cover test), stereopsis (Titmus Fly SO-001 StereoTest), binocular fusion test (a Worth 4 dot test) and gender of participants with amblyopia are provided in Table 1. All participants were tested with their best-corrected visual acuity for the dichoptic letter chart protocol although we attempted to carry out the assessment procedure in a natural viewing setting in which subjects’ binocular fusion was controlled by the high contrast frame of the display and the room’s spatial structure in the peripheral visual field. We did not attempt to correct binocular image alignment or strabismus. Note that we used the term “strong eye” to refer to the dominant eye for strabismus or normal vision. The strong eye was determined by clinical assessments (either visual acuity, binocular fusion test or both for amblyopia) or finger pointing task (for controls).

### Stimuli and Apparatus

**Figure 5:**
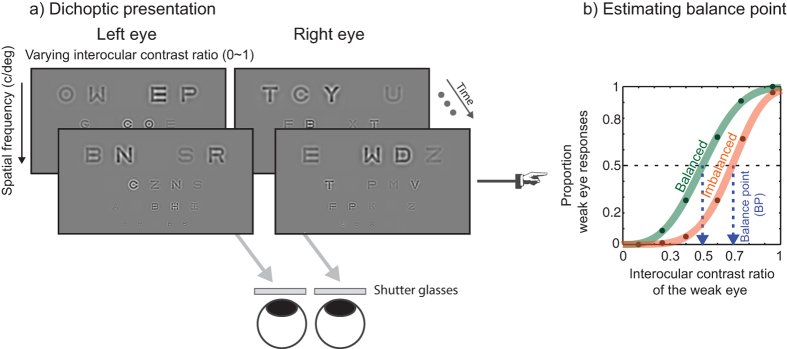
Schematic diagrams of stimulus and task. **(a)** Dichoptic stimulus presentation. Test stimuli were band-pass filtered Sloan letters with peak spatial-frequencies of 0.5, 1.5, 2.5, and 5.0 c/deg. Letters had a layout similar to the ETDRS acuity chart (4 rows of decreasing letter size by 5 columns of varying letter contrast) on a gray background. Each row contained letters at a single SF and a range of interocular contrast ratios (achieved by presenting two different letter charts to each eye using stereo-shutter glasses). Participants were instructed to read the chart aloud from left-to-right, from top-to-bottom line. The relative contrast of the letter in each eye was adjusted across several charts to determine the interocular balance point (BP). The BP is the interocular contrast-ratio required for participants to report the letter presented to either eye with equal probability. **(b)** Estimating BP from a psychometric function: An example showing the proportion of weak eye responses versus interocular contrast ratio (note that the proportion of *strong eye* responses are simply 1 minus the proportion of weak eye responses). Proportion weak eye responses were measured as a function of interocular contrast ratio and resulting data fit with a Weibull function to derive a BP: the interocular contrast ratio yielding 50% identification for each eye (black dashed line). The solid line is the best fit of the function. The dotted arrow line (blue) indicates the estimated BP. The green curve demonstrates hypothetical data from a normally sighted individual whose binocular vision is equated, i.e. BP ≈ 0.5. The orange curve shows hypothetical data from an individual whose weak eye is suppressed, resulting in a BP > 0.5.

The 26 Sloan font letters of the English alphabet were used for measuring binocular imbalance (Fig. 5a). Test letters were spatially band-pass filtered with a cosine log filter^71^ with peak object SF of 3 cycles per letter (c/letter). The filter has a bandwidth (full-width at half-height) of 1-octave and is radially symmetrical in the log-frequency domain. The retinal SF of the test letters ranged from 0.5 to 5 cycles per degree (c/deg) at the viewing distance of 57 cm. This was achieved by fixing the object SF at 3 c/letter and varying image sizes (0.6°, 1.2°, 2°, and 6°). The object SF of 3 c/letter was chosen in light of previous findings showing that the critical (optimal) spatial-frequency for recognizing letter lies between 1 and 7 c/letter depending on letter size^72^,^73^,^74^,^75^,^76^,^77^. We chose letter stimuli because of the following reasons: 1) people are highly familiar with letter acuity charts such as Snellen or HOTV chart, which makes the test much easier to administer; 2) ten Sloan band-pass filtered letters have been successfully used in previous studies measuring contrast sensitivity function in normal or visually impaired individuals^78^,^79^,^80^. Although legibility and confusions among the unfiltered Sloan optotypes have been well-characterized^81^, equivalent data for band-pass filtered Sloan optotypes are not yet available. Nevertheless, the randomization across conditions in our study should not introduce any systematic biases in the BP data; and 3) Given the fact that a subset of amblyopia show more acuity deficits in optotype stimuli than grating stimuli^8^,^82^, it appears to be advantageous to use optotype stimuli for measuring amblyopic deficits. Our letter stimuli might not be readily accessible to non-literate children or non-native English speakers, especially when we consider the importance of early detection and diagnosis in treating amblyopic vision. For this reason, we have implemented our current dichoptic chart using band-pass filtered number optotypes^83^ and Lea Symbols^84^ and the validity of the test with these alternate optotypes is currently under investigation.

Test letters were displayed on a uniform gray background (60 cd/m^2^) with varying contrasts defined as Michelson contrast. The stimuli were generated and controlled using MATLAB (version 7.9) with Psychophysics Toolbox extensions^85^,^86^ for Windows 7, running on a PC desktop computer (model: HP Pavilion). Stimuli were presented on a liquid crystal display monitor (model: Asus VS278H-E; refresh rate: 120 Hz; resolution: 1920 × 1080) with the maximum luminance of 250 cd/m^2^. Stimuli were rendered with 10.8-bit grayscale levels using the bit-stealing method^87^. The monitor was calibrated using a spectrophotometer (model: Photo Research SpectraScan 655) and linearized. Participants wore stereo-shutter glasses (nVidia Corp., Santa Clara, CA) running at 60 Hz frame rate per eye. The glasses reduced the luminance of the display monitor by a factor of 3 and had low crosstalk between the two lenses (<5%).

### Procedure

As shown in Fig. 5a, the letters were arranged in a layout similar to the ETDRS acuity chart^88^,^89^ (4 rows of decreasing letter size by 5 columns of varying letter contrast). To minimize any potential confusion, the same letter could not appear twice in the same row. A different letter chart was presented to the weak and strong eyes of an observer via stereo-shutter glasses. At each position, the identity and interocular contrast-ratio of the letter on each chart differed while the SF content of the letter remained the same. The sum of interocular contrast ratio across the two eyes was fixed at 100% contrast. Thus, if the contrast in the weak eye were 70%, the contrast in the strong eye would be 30% and vice versa. Interocular contrast ratio was randomized across 5 letter slots in each row to avoid any ascending or descending pattern of contrast arrangement, which might have biased participants’ response.

Participants freely viewed the chart and were instructed to read the chart-letters aloud, from left-to-right, and from top line to bottom line, as quickly and accurately as possible. If participants experience binocular rivalry which may occur when the interocular contrast ratio reaches a subject’s balance point, they were instructed to report the more dominant percept (whichever comes first or whichever appears to be a stronger percept). If subjects happened to report a third letter identity (e.g., as a result of a piece-meal rivalry, which is a common and well-documented percept in binocular rivalry^41^,^42^,^43^, although it was rare in our study), this response was considered as incorrect response for both eyes, so that it did not bias toward either one of the eyes.

The participants read the perceived letter aloud and responses were recorded by the experimenter via a keyboard. Completion of the present chart initiated a subsequent chart marked by an auditory beep. The relative contrast of the letter in each eye was determined via an adaptive procedure as follows: For a given chart, proportion correct recognition was computed at each interocular contrast ratio (each letter slot, 5 slots per line), which was used to estimate the *balance* point (BP) between the two eyes for each SF (each line in the chart). The BP was defined as the interocular contrast-ratio yielding letter recognition in each eye with equal probability (50%). An ongoing estimate of BP was updated after each letter chart with accumulating number of trials, and the updated BP was used as a basis for determining the range of contrast ratios for a subsequent chart. Therefore, over several charts (e.g., 12 charts), the range of interocular contrast-ratio converged to an estimated BP so that more testing points were sampled near the estimated BP. The updating process was achieved via an in-house adaptive algorithm in conjunction with QUEST^90^. This algorithm takes advantage of the outcome of previous trials to determine the range of interocular contrast ratio and the space between adjacent test points that deliver maximum information about the subject’s BP for a subsequent trial. This update procedure was simultaneously, but independently computed for each SF arranged in each row. Overall, at least 60 data points (12 charts × 5 contrast ratios) were accumulated to estimate the BP for each spatial frequency. Participants were given a few practice trials before the experimental test to make sure they fully understood the task and procedure. A chin-rest was used to maintain a constant viewing distance. The session lasted approximately 6 ~ 7 mins. We repeated the session twice to estimate test-retest reliability for a subset of participants.

Having obtained letter identification data at varying contrast ratios via the foregoing adaptive procedure, we computed the final BP at which the two eyes’ inputs reach equilibrium by fitting the psychometric function to the data. Psychometric functions of percent weak eye identity responses versus interocular contrast ratio were created by fitting the data with Weibull functions^91^,^92^ as shown in Fig. 5b. The curve fits were achieved for each subject using the simplex search method^93^ to minimize the weighted residual sum of squares. The reciprocal of the variance of each data point (1/σ^2^) was used to weight the fits. The BP was based on the estimated 50% correct point on the psychometric function for each SF. For example, the BP of 0.5 indicates that the letter in each eye was reported with equal probability when the contrast in the weak eye was 50% and the contrast in the strong eye was 50% (Fig. 5b), suggesting the input signal strengths of the two eyes are equated. On the other hand, when the BP of 0.8 means that the letter in each eye was reported with equal probability when the contrast in the weak eye was 80% and the contrast in the strong eye was 20%. Thus, the larger the BP is (i.e., a rightward shift of the psychometric function), the more attenuated or suppressed is the input signal of the weak eye.

To examine if there are any significant differences in BP among tested SFs and between amblyopes and normal controls, data were analyzed using a repeated measures analysis of variance (ANOVA) and Tukey’s HSD pairwise comparison test. We confirmed that the data were normally-distributed with a Quantile-Quantile plot. Test-retest reliability was evaluated using i) Pearson’s Product Moment Correlation coefficient (*r*) and ii) Bland & Altman difference plot^53^ in which difference values between the two measurements (i.e., 1^st^ test − 2^nd^ test) are plotted as a function of mean values of the two (i.e., (1^st^ test + 2^nd^ test)/2) for each subject. The test-retest variability was estimated as 95% confidence interval limits of agreement (mean difference between measures ± 1.96 SD). Sixteen out of twenty four subjects participated in the test-retest reliability test.

## Additional Information

**How to cite this article**: Kwon, M.Y. *et al.* Spatial-frequency dependent binocular imbalance in amblyopia. *Sci. Rep.* **5**, 17181; doi: 10.1038/srep17181 (2015).

## Figures and Tables

**Table 1:** Characteristics for the amblyopic participants.

SubID (gender)	Age (yrs)	Type	Visual Acuity (logMAR)		Refractive Error		Angular Deviation (Δ)	Stereoacuity (")	Suppression
			OD	OS	OD	OS			
A1 (F)	15	Anisometropic	−0.20	0.20	plano	2.5	Ortho	400	Suppression
A2 (F)	22	Anisometropic	0.04	–0.20	+2.5	plano	Ortho	900	Suppression
A3 (M)	24	Anisometropic	−0.20	0.12	+2.25	+7.25/−0.75 × 30	Ortho	900	Suppression
A4 (F)	25	Anisometropic	0.10	–0.14	+4/−1.25 × 110	plano	Ortho	500	Intermittent Suppression
A5 (F)	20	Meridional, Anisometropic (Exophoria)	0.09	0.50	−6.65/ + 3.75 × 80	−7.25/ + 4 × 95	4	400	Intermittent Suppression
A6 (F)	24	Mixed (Esotropia)	0.00	0.10	−3.25/−3.5 × 175	−5.75/−3.75 × 13	12	900	Suppression
A7 (F)	25	Anisometropic	0.10	–0.10	4.75/−1.75 × 70	1/0.25 × 75	ortho	400	Suppression
A8 (F)	22	Anisometropic	0.00	–0.20	2.5	plano	ortho	900	Suppression
A9 (M)	23	Anisometropic	−0.3	0.10	plano	1/−1.25 × 70	ortho	900	Suppression
A10 (F)	25	Anisometropic	−0.10	0.00	2	6.75/−0.75 × 40	ortho	900	Suppression
A11 (F)	24	Anisometropic	0.00	0.10	3/−3.25 × 170	4.25/−3.25 × 170	ortho	400	Suppression
A12 (M)	48	Strabismic (Esotropia)	−0.08	0.20	+0.75	+1.25	22	900	Suppression

Note that Δ: Prism diopter, ": Arcsecond, OD: Right eye, OS: Left eye.
